# Genetic evidence for plural introduction pathways of the invasive weed Paterson’s curse (*Echium plantagineum* L.) to southern Australia

**DOI:** 10.1371/journal.pone.0222696

**Published:** 2019-09-19

**Authors:** Xiaocheng Zhu, David Gopurenko, Miguel Serrano, Mark A. Spencer, Petrus J. Pieterse, Dominik Skoneczny, Brendan J. Lepschi, Manuel J. Reigosa, Geoff M. Gurr, Ragan M. Callaway, Leslie A. Weston

**Affiliations:** 1 Graham Centre for Agricultural Innovation (Charles Sturt University and NSW Department of Primary Industries), Charles Sturt University, Wagga Wagga, Australia; 2 NSW Department of Primary Industries, Wagga Wagga Agricultural Institute, Wagga Wagga, Australia; 3 Department of Botany, Faculty of Pharmacy, University of Santiago de Compostela, Santiago de Compostela, Spain; 4 Department of Life Sciences, Natural History Museum, London, United Kingdom; 5 Department of Agronomy, Stellenbosch University, Private bag X1, Matieland, South Africa; 6 Department of Biochemistry and Molecular Biology, University of Melbourne, Melbourne, Australia; 7 Australian National Herbarium, Centre for Australian National Biodiversity Research, Canberra, Australia; 8 Department of Plant Biology and Soil Science, Faculty of Biology, University of Vigo, Vigo, Pontevedra, Spain; 9 Division of Biological Sciences, University of Montana, Missoula, Montana, United States of America; Universidade da Coruna, SPAIN

## Abstract

Paterson’s curse (*Echium plantagineum* L. (Boraginaceae)), is an herbaceous annual native to Western Europe and northwest Africa. It has been recorded in Australia since the 1800’s and is now a major weed in pastures and rangelands, but its introduction history is poorly understood. An understanding of its invasion pathway and subsequent genetic structure is critical to the successful introduction of biological control agents and for provision of informed decisions for plant biosecurity efforts. We sampled *E*. *plantagineum* in its native (Iberian Peninsula), non-native (UK) and invaded ranges (Australia and South Africa) and analysed three chloroplast gene regions. Considerable genetic diversity was found among *E*. *plantagineum* in Australia, suggesting a complex introduction history. Fourteen haplotypes were identified globally, 10 of which were co-present in Australia and South Africa, indicating South Africa as an important source population, likely through contamination of traded goods or livestock. Haplotype 4 was most abundant in Australia (43%), and in historical and contemporary UK populations (80%), but scarce elsewhere (< 17%), suggesting that ornamental and/or other introductions from genetically impoverished UK sources were also important. Collectively, genetic evidence and historical records indicate *E*. *plantagineum* in southern Australia exists as an admixture that is likely derived from introduced source populations in both the UK and South Africa.

## Introduction

Invasive species can be defined as those that are distributed beyond their native ranges and pose a significant negative impact on indigenous ecosystems, public health or agricultural production [1, 2]. Annual losses of USD $1.4 trillion were estimated to be associated with global biological invasions [1]. Invasion success is influenced by the number of introduction events, post-introduction distribution vectors and associated impacts of introduced populations on indigenous ecosystems [1, 2].

Recent investigations of invasive species have focused on anthropogenic activities that directly or indirectly drive global species dispersal [2–4], and often involve complex multi-source pathways of introduction and/or repeated introductions [5, 6]. In contrast, natural invasions are frequently characterised by range expansion over a range of geographical and temporal scales [2].

Accurate reconstruction of invasion histories is also fundamental to the understanding of biological invasions [2]. Successful reconstruction demonstrates the invasion route and therefore assists in the determination of the genetic composition of both past and current invasive populations [7]. Invasion history reconstruction is also critical for developing biological control approaches, prediction of future introductions and determination of both economic and ecological impacts of invasive species [2, 6]. In some cases, introduction routes can be directly reconstructed from historical evidence [8, 9]. However, in most cases, this is not possible due to the antiquity of key events and the limited availability of biological records, such as herbarium specimens. This is especially true of invasions associated with accidental introduction [7]. Therefore, indirect methods evaluating population genetic structure provide an alternative means of reconstructing introduction histories [10–13].

Genetic analyses using molecular markers can provide detailed information on the history of biological invasions [14]. Genotyping using simple sequence repeats, Sanger sequencing [11, 15] and Next-Generation Sequencing [10] can be used to determine taxonomic identity, source(s) of introduction, genetic bottlenecks, national and international dispersal vectors, and predict the number of introduction events (single or multiple) [2]. For example, microsatellite and chloroplast DNA sequence analyses of globally collected samples of *Silene latifolia* Poir. suggested that this species originated from Europe and was introduced on multiple occasions to the Great Lakes region (USA) via western North America [11].

Our research has focused on the annual or biennial invasive weed species *Echium plantagineum* L. (Boraginaceae), also known as Paterson’s curse, a native of the Mediterranean region and adjacent areas of Atlantic western Europe. It is predominantly distributed across the western half of the Iberian Peninsula [16]. The first reliable report of cultivated *E*. *plantagineum* in the UK was in 1776 [17]. However, it is possible that *E*. *plantagineum* was present in the UK earlier than 1776, because the plant was confused with other members of the Boraginaceae by pre-19th century English botanists [18]. It is now only found in one small population of <10 ha in Cornwall. More recently, it has occurred as an escape from ‘wildflower’ seed mixes used to attract bees and other invertebrates. It is also found in the Channel Islands where it may be native and has been known of since the 17^th^ century. *Echium plantagineum* has also been introduced to South Africa through shipments of contaminated stock feed from Europe, but the date of introduction is unknown [19]. Currently, this species is considered an invasive weed in Australia [20], naturalised in South Africa along the south eastern coastal area [21] and found infrequently in the USA [22], central and Eastern Europe [16] and New Zealand [23].

In Australia, where *E*. *plantagineum* covers now more than 30 M ha, the species successfully outcompetes native pasture grasses, and through adaptation to climatic extremes due to its drought tolerance and fecundity[20]. The exceptional spread of *E*. *plantagineum* across key temperate agricultural zones has resulted in over A$250M in losses to the Australian meat and wool industries [24]. *Echium plantagineum* produces toxic pyrrolizidine alkaloids in its shoots [25, 26] and bioactive (and potentially allelopathic) shikonins in its roots [27, 28]. It is also known as Salvation Jane, as it is often the only forb remaining green in drought-stricken paddocks, thereby supporting grazing despite its toxicity. *Echium plantagineum* is not considered an economically important weed outside of Australia [23].

Previous studies of *E*. *plantagineum* revealed that it exhibits considerable genetic diversity within Australia [20, 23, 29]. These findings align well with available historical records noting its first and most recent location records [18, 30]. High levels of genetic diversity in Australian populations were detected using isozyme analysis [29] and a more recent sequence analysis [20] found regionally specific haplotypes and distinct genetic structure in populations from southern Australia, suggesting the possibility of separate multiple introduction events [20]. Herbarium records from the 1800’s suggest that *E*. *plantagineum* was likely introduced to Australia from the UK as an ornamental species on numerous occasions since 1843 by its propagation in public and private gardens across New South Wales (NSW), Victoria (VIC), South Australia (SA) and Tasmania (TAS) [30].

Despite the economic and cultural significance of this weed across southern Australia, the key vectors for its international dispersal remain unknown. Dispersal in the UK, South Africa and Australia is most frequently associated with anthropogenic activity through introduction as an ornamental or by accident through contaminated agricultural products [18, 19, 23, 30]. Since 1788, Australia has regularly imported Merino sheep from South Africa and western Europe [31]. Given that Australia has a long history of livestock, feed and fodder importation from the countries which harbour *E*. *plantagineum*, it is highly likely that accidental introduction of this species as seed contaminant in either feed and fodder or on the fleeces of live animals contributed to the rapid spread of this weed across pastoral Australia in the 1800s.

Genetic analysis using molecular approaches along with historical record evaluation is critical to develop a clear understanding of the invasion history of *E*. *plantagineum* in Australia. We therefore focused on several polymorphic chloroplast gene regions to gain a deeper insight into the genetic structure of this species by collecting fresh plant material as well as herbarium specimens in both its native (Iberian Peninsula) and introduced ranges (UK, Australia and South Africa) for advanced analysis. Sequencing data as well as historical references were further analysed to reconstruct the introduction history of *E*. *plantagineum* in Australia. Given the frequent and longstanding importation of Merino sheep from western Europe and the UK to Australia [31], we hypothesized that, in addition to the notable and previously described ornamental introductions, accidental introduction associated with livestock transport also further enabled invasion of *E*. *plantagineum* in Australia.

## Materials and methods

### Plant material and DNA extraction

**Fig 1:**
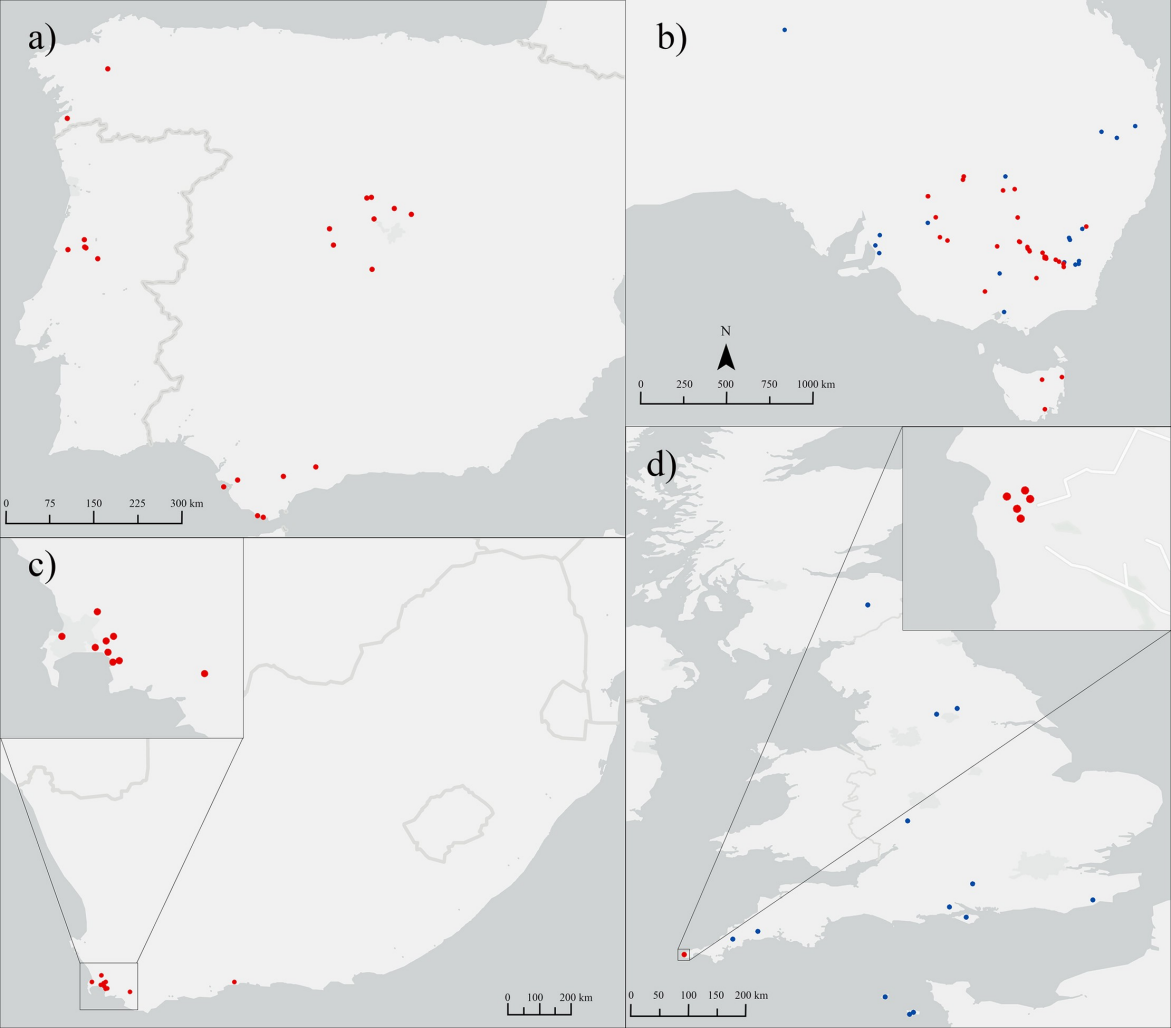
Distribution of sampling locations across Iberian Peninsula (a), Australia (b), South Africa (c) and the UK (d). Four samples from Western Australia are not shown on the map. Blue indicates samples donated from Australian National Herbarium or British National History Museum. Maps were created using ArcGIS software by Esri. The base map is sourced from Esri. "Light Gray Canvas" [basemap]. April 25, 2018. https://www.arcgis.com/home/item.html?id=ee8678f599f64ec0a8ffbfd5c429c896. (June 9, 2019).

Samples were collected from non-native populations in Australia and potential source populations across the Iberian Peninsula, South Africa and in the UK. As limited global distribution of *E*. *plantagineum* exists in areas other than Australia, the UK and southern Europe, populations other than those collected by our team were not considered in this study. In total, 253 *E*. *plantagineum* specimens were sampled and genetically analysed (Fig 1 and S1 Table). These included specimens from the native range in the Iberian Peninsula (N = 47), and from invasive populations in South Africa (N = 50), the UK (25), and Australia (N = 131, including previously reported samples [20] listed under GenBank accession numbers KX012236-KX012622). Samples obtained included vouchered specimens available from the Australian National Herbarium (Canberra, N = 14) and the Natural History Museum (London, N = 15). Fresh specimens (N = 224) sampled as healthy, non-senesced leaf tissue were obtained across the Iberian Peninsula, southern Australia, the Cape of Good Hope in South Africa and the UK. In Australia, samples obtained from all major invaded regions and states, but the greatest number of samples were obtained from the region of greatest infestation, Southern Australia. We sampled native *E*. *plantagineum* populations in the Iberian Peninsula across a wide range of climatic conditions and soil types, where considerable native diversity is likely to be found. Although the species proliferates on acidic soils [16], which explains its predominantly western Iberian range, we sought to include some rare populations from limestone or mafic areas in the analysis. Similarly, the Iberian samples encompass most of the environmental niche breadth of the species, through sampling in different bioclimates [32] in the Atlantic and Mediterranean biogeographic regions of the Iberian Peninsula. Native samples were subsequently obtained from north-western Iberia, central Portugal, the northern central Spanish plateau, the southern central Spanish plateau and south-western Iberia including the Atlantic and Mediterranean areas of Andalusia. In the UK, plants were sampled from a coastal location along the cliffs of Cornwall, the only known wild and permanent population of *E*. *plantagineum* in the UK. Sampling in South Africa was focused on the Cape of Good Hope, an important port for trade between Europe, Asia and Australia between the 15th and 20th centuries and of historical relevance in this study [19, 21, 31]. Upon collection, samples were preserved in 100% ethanol or silica gel until DNA extraction. Genomic DNA isolation was conducted using a gene robot (Corbett Research 1820 X-tractor) as previously described [33]. We note that an additional N = 25 vouchered specimen samples listed in S2 Table failed to PCR amplify and were excluded from the total sample set reported here.

### PCR amplification and sequencing

Previous research conducted to determine optimal gene regions for the discrimination of population genetic structure in *E*. *plantagineum* revealed that three chloroplast gene regions (*trnH*-*psbA* spacer, *trnL* intron and *trnL*-*trnF* spacer) were useful targets and more informative than nuclear ribosomal targets [34]. Following PCR amplification, universal primers [35, 36] were selected for PCR reactions (S2 Table). Specifically, 17 bp M13 vector sequence tails were incorporated at the 5’-ends of each primer to simplify downstream sequencing. PCR was performed in 15 μL reactions. The final PCR reaction contained 1 μL DNA template, 1X PCR buffer, 3 mM MgCl_2_, 200 μM dNTPs, 1.5 μM of each primer and 0.4 U Platinum Taq polymerase (Invitrogen, Australia). Reactions were conducted with a PCR instrument (Eppendorf Mastercycler, Eppendorf, Australia). Following initial denaturation of two minutes at 94 ºC, 35 cycles of 30 seconds at 94 ºC, 30 seconds at annealing temperature (S2 Table) and 1 min extension at 72 ºC was performed, followed by a final extension at 72 ºC. Selected amplicons were sequenced by the Australian Genome Research Facility (AGRF, Brisbane, Australia).

### Data analysis

Sequencing quality was validated using the software package SeqMan Pro 8.1 (DNASTAR, Inc.), and sequences were aligned using Clustal W [37] through Bioedit [38], followed by manual adjustment. A homopolymeric region (polyA and polyT) present in the *trn*L intron resulted in low sequencing quality of a 25 bp portion, which was later discarded from the sequence alignment for further analysis. Alignments were truncated to 284, 488 and 399 bp for *trnH*-*psbA* spacer, *trnL* intron and *trnL*-*trnF* spacer, respectively. The chloroplast regions were later concatenated as a 1,133 bp alignment following recoding of indels and inversions as 5th character state mutations, and exclusion of a 25 bp homo-polymeric region present in the *trnL* intron (S1 Appendix) [39, 40]. Alignments of the three linked chloroplast DNA regions were concatenated using FABOX software [41]. All novel sequences reported in this study were deposited in the GenBank database (accession numbers: MG597841—MG598306).

Nucleotide (π) and haplotype (*h*) diversity [42] at populations, and pair-wise genetic structure between populations (*F*_***ST***_) based on haplotype diversity [43] were calculated using ARLEQUIN version 3.5 [44]. Significance of pairwise *F*_*ST*_ estimates was determined by permutation (10,000 replicates) as implemented in ARLEQUIN. Population genetic structure between historical (since 1800s) and recent (2016) samples collected from the UK was also estimated. Standardized haplotype richness was calculated for each population after rarefaction to the UK samples size of n = 25 using Contrib 1.40 [45, 46]. Genealogical relationships among haplotypes were inferred by 95% parsimony network analysis using TCS software version 1.21 [47].

## Results

### Sequencing data and haplotypes

**Fig 2:**
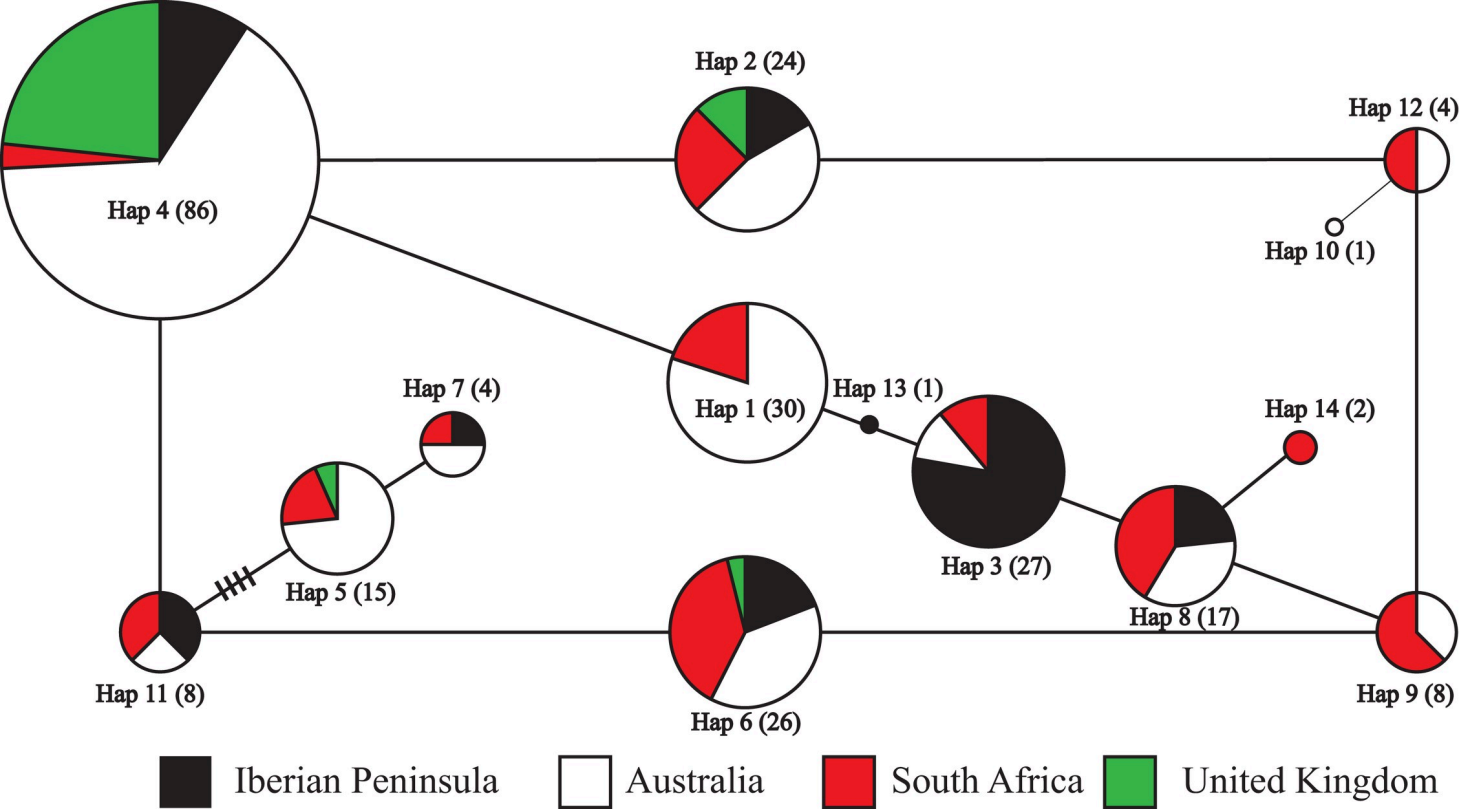
Parsimony (95%) networks showing the evolutionary relationships between the 14 haplotypes found in *Echium plantagineum* collected from the Iberian Peninsula, Australia, South Africa and the United Kingdom. Indels were included as a 5th character in the analysis. Haplotypes (Hap) are represented by circles and labelled as per S1 Table. Haplotype frequency is denoted by circle size and the parentheses enclosed the number of individuals found for each haplotype. Lines between haplotypes indicate a single mutation, while cross bars on the line represent hypothetical unsampled haplotypes.

In total, 14 haplotypes were revealed following concatenation of the chloroplast regions, with 8, 12, 12 and 4 haplotypes observed in the Iberian Peninsula, Australia, South Africa and UK, respectively (S3 Table). The 95% parsimony network analysis (Fig 2) indicated shallow phylogenetic relationships among the haplotypes owing to paucity and/ or homoplasy of the variable sites selected for analysis in this study.

**Fig 3:**
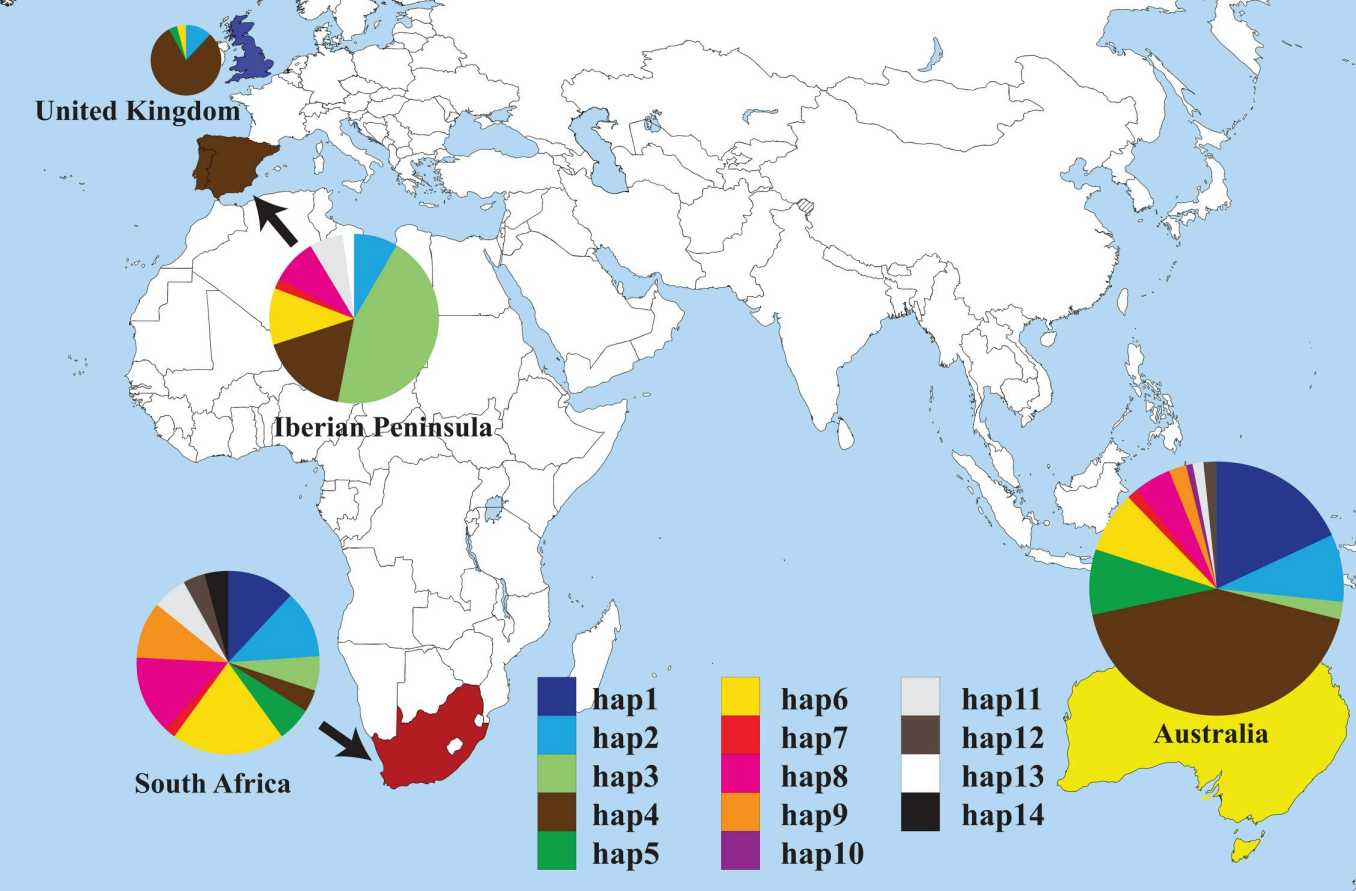
Distribution of haplotypes of *Echium plantagineum* in the Iberian Peninsula, Australia, South Africa and the United Kingdom. Circle size is related to the number of successfully sequenced samples in each region. Haplotypes are labelled as per S1 Table. This map is a derivative of *“*A large blank world map with oceans marked in blue.PNG*”* sourced from Wikimedia Commons, used under Public Domain license and modified using Adobe Illustrator CS5.

Region-specific haplotypes (Hap 13, 10 and 14) were found in the Iberian Peninsula, Australia and South Africa, respectively (Figs 2 and 3, S3 Table). Most Australian haplotypes were also recovered in the Iberian Peninsula (7 out of 12) and South Africa (11 out of 12). The distribution of haplotypes in each population is represented in Fig 3. Australian *E*. *plantagineum* was dominated by the presence of Hap 4 detected in 43% of Australian samples. Hap 4 was also highly abundant in the UK (80%), but much less abundant in the Iberian Peninsula (17%) and in South Africa (4%). On the Iberian Peninsula, this haplotype was mainly found in southern Spain (7 out of 8). In contrast, Hap 3 was the dominant genotype observed in the Iberian Peninsula (45%), and the distribution of all 12 haplotypes found in South Africa was relatively uniform (Fig 3). High level haplotype diversity (*h*) in *E*. *plantagineum* was found among samples collected from the Iberian Peninsula (0.7567), Australia (0.7657) and particularly in South Africa (0.9037); UK samples demonstrated much lower haplotype diversity (0.3567) (Table 1). Standardized estimates of haplotype richness after rarefaction (n = 25, Table 1) revealed similar trends with high haplotype richness in Iberian Peninsula (5.87), Australia (6.61) and South Africa (9.36) and lower richness in the UK (3.00). Low levels of nucleotide diversity (*π*) were observed among samples in all regions (Table 1). *Echium plantagineum* collected in the UK showed the lowest nucleotide diversity at 0.0008, while the highest nucleotide diversity was observed in South African samples (0.0023).

**Table 1:** Haplotype (*h*), nucleotide (*π*) diversity and adjusted allelic richness of *Echium plantagineum* populations from the Iberian Peninsula (IP, native range), Australia, South Africa and the UK (non-native ranges).

Populations	*n*^a^	*h*	*π*	r(25)^b^
IP	47	0.7567 ± 0.0514	0.0017 ± 0.0011	5.87
Australia	131	0.7657 ± 0.0297	0.0020 ± 0.0012	6.61
South Africa	50	0.9037 ± 0.0177	0.0023 ± 0.0014	9.36
UK	25	0.3567 ± 0.1150	0.0008 ± 0.0006	3.00

^a^ *n*: number of samples that were successfully sequenced in *trnL* intron, *trnL*-*trnF* spacer and *trnH*-*psbA* spacer.

^b^ r(25): adjusted allelic richness after rarefaction to the smallest population size in the UK (n = 25) using Contrib 1.40 [45]

### Genetic structure analysis

All pairwise estimates of population genetic structure (*F*_*ST*_) were significant (*P* < 0.05; Table 2). Shallow but significant population genetic structure was evident (Table 2) between Australia and UK (pairwise *F*_*ST*_ = 0.061; *P* < 0.05), and also evident between the Iberian Peninsula and South Africa (pairwise *F*_*ST*_ = 0.061; *P* < 0.05). In contrast, higher levels of genetic structure were evident in comparisons between reciprocal populations of these two groups (pairwise *F*_*ST*_ > 0.125; *P* < 0.001).

**Table 2:** Pairwise comparison (shown as pairwise *F*_*ST*_ value) of genetic structure between *Echium plantagineum* populations. Genetic structure is significant at * *P* < 0.05 and *** *P* < 0.001.

Populations	Australia	UK	South Africa
UK	0.061*		
South Africa	0.192***	0.408***	
Iberian Peninsula	0.126***	0.313***	0.061*

### Comparison between historical and recent UK *E*. *plantagineum*

Vouchered specimens at the Natural History Museum, London dating back to the 1800’s provided an opportunity for comparison of historical (N = 15) and recent (N = 10) genetic structure of *E*. *plantagineum* in the UK. Four and two haplotypes were found among historical and recent UK samples, respectively. Haplotype 4 accounted for 80% of all haplotypes among both the historical and recent UK *E*. *plantagineum* sampling efforts. No significant difference in structure was found between the temporal sampling efforts (pairwise *F*_*ST*_ = -0.0491; *P* = 0.784).

## Discussion

Our results demonstrate a relatively complex pathway of introduction of *E*. *plantagineum* to Australia, through incorporating considerable numbers of field and herbarium samples spanning nearly two centuries. Sampled populations from the Iberian native range were representative of a significant range of soil types and climatic variation across the peninsula. We were able to sample an important population in the UK where the species is currently rare and endangered [48]. We also retrieved historical specimens from herbarium in the UK for additional analyses. Significantly, nearly all haplotypes found in Australia were also recovered from sampling conducted in the Cape of Good Hope region in South Africa (S3 Table). However, further statistical analysis showed that Australian *E*. *plantagineum* was more closely aligned with populations collected in the UK, likely due to the prevalence of Hap 4 in both Australia and the UK, in contrast to those collected in the Iberian Peninsula and South Africa (Table 2).

### Complex introduction of *E*. *plantagineum* in Australia

A high level of genetic diversity in invaded ranges is typically associated with multiple proactive and accidental introduction events. In this case, plants imported as ornamentals from the UK as referred to in historical records, as well as likely propagule contamination associated with movement of feed, fodder or livestock transported from Western Europe to Australia via South Africa. Both scenarios are likely responsible for the repeated and prolonged introduction of this species to the Australian continent over the last two centuries.

The relatively high frequency of Hap 4 in both Australia and the UK suggests the possibility that Hap 4 may have preferentially been selected as a more successful and subsequently invasive biotype that served as the bridging population in Australia, described in the literature as the bridgehead effect [49]. The bridgehead effect has been observed in association with the introduction of numerous other invasive species including plants [10, 11], insects [49, 50] and pathogens [51]. Remarkably, Hap 4 represented only 4% of all haplotypes noted in South Africa. Therefore, it is difficult to envisage how selection for Hap 4 occurred independently in Australia and not elsewhere, particularly as South Africa’s Cape of Good Hope region has a somewhat similar Mediterranean climate to that of heavily infested regions in Australia. Thus, it is most likely that the initial introduction of *E*. *plantagineum* to Australia was associated with a genetically impoverished UK population, described in historical records as the potential origin of *E*. *plantagineum*, as an ornamental plant in the 1840’s, in the State of New South Wales, Australia [23]. Additionally, the accidental introduction associated with livestock importation likely occurred, thereby resulting in Hap 4 as the dominant haplotype in southern Australia. Subsequent multiple introductions of *E*. *plantagineum* from genetically diverse populations in South Africa and Western Europe potentially also contributed to increased genetic diversity in Australian *E*. *plantagineum* over time (S1 Fig).

For invasive plants, anthropogenic activities frequently remove long-standing physical barriers to species migration, and major trade routes also encourage movement of potentially invasive plants from their native range into non-native regions. In its native range, *E*. *plantagineum* is frequently associated with livestock movement in disturbed pastures [23, 52]. Historical records from the 19^th^ and 20^th^ century suggest that the introduction and spread of *E*. *plantagineum* in Australia occurred mainly through seed contaminants in feed and fodder [30]. Australia, further described as a country “riding on the sheep’s back”, has a long history of importation of the Merino breed from several countries including Spain, where the Merino originated. In 1788, Merinos reared in South Africa were first introduced to Australia, followed later by multiple introductions of these and other breeds from the UK and Spain via South Africa [31]. As most of the vessels arriving in Australia in the 19^th^ century originated from the UK [53], there were ample opportunities for the dominant Hap 4 in the UK to become prevalent in Australia.

*Echium plantagineum* is also very likely to have been directly introduced to Australia from South Africa as extended periods of rest and restocking occurred when vessels were laying over in South Africa [54]. We observed that nearly all Australian haplotypes were detected in samples collected from the major site of infestation near the Cape of Good Hope in South Africa (Fig 3 and S3 Table). Given the relative rarity of the plant in the UK [48], as noted in reports of infrequent sightings across the UK from the 1700s onwards (Mark A. Spencer, unpublished data), and the limited genetic diversity observed in both historic samples and those from the sole remaining wild UK population in Cornwall, it is highly likely that populations in South Africa were introduced from sources other than the UK.

Since 1488, the Cape region of South Africa is noted as the most important seaport connecting Europe and Asia, and has long been a reservoir for numerous invasive European plants [55] that are also noxious species in Australia including *E*. *plantagineum*, blackberry (*Rubus fruticosus* agg.) and gorse (*Ulex europaeus* L.). The majority of vessels laying over in the Cape region of South Africa in the 16^th^ to 17^th^ century originated from areas in western Europe [54] that were in close proximity to native populations of *E*. *plantagineum*. Given the considerable time frame for active maritime transport from Europe to South Africa, even the rarest native haplotypes of *E*. *plantagineum* could have been successfully introduced to South Africa. Thus, the high level of genetic diversity and number of haplotypes found in South Africa, and the relative similarity in genetic structure between South African and the Iberian Peninsula, both support the hypothesis of transport of *E*. *plantagineum* from its native range to South Africa over a prolonged period of time. Despite widespread geographical sampling across the Iberian Peninsula, six haplotypes presented in the non-native ranges (Hap 1, 5, 9, 10, 12 and 14) were not detected. This could reflect low current frequencies of these six haplotypes in the native ranges and inadequate sampling intensity, or subsequent extinction of the haplotypes. Therefore, more intensive sampling could help to resolve this question. Additional studies focusing on the species phylogeography in the native range, encompassing a wider geographical extent in the native or anciently introduced areas in the Mediterranean basin, might also assist in detection of possible source locations. The presence of these six haplotypes in introduced ranges further suggests that multiple founder events associated with transport from South Africa may have allowed for the fortuitous entry of low frequency haplotypes from the native range and subsequently their greater representation in introduced localities.

Similar to *E*. *plantagineum*, complex introduction pathways of invasive species have been found for many other plant invaders to Australia such as *Lantana camara* L. [56], *R*. *fruticosus* [57] and *U*. *europaeus* [58]. Interestingly, all of these species are currently naturalized in South Africa. *Rubus fruticosus* and *U*. *europaeus*, which are native to western Europe, were both proactively introduced to Australia in 1800s [57, 59]. *Lantana camara* which is native to central America, was first introduced to western Europe and then to Australia via South Africa [60]. Together with the introduction of *E*. *plantagineum*, it is clear that South Africa has played a critical role in Australian plant invasions.

Previous studies showed that some invasive plants developed more competitive traits including greater fertility[61] and higher abundance of chemical weapons[62]. The importance of the role of phenotypic plasticity for successful invasions is exceeded by the development of novel heritable traits in the invasive population [63, 64] that ultimately result in more competitive invaders [65]. Therefore, studies of the pathway of plant introduction are critical for prediction and prevention of future introductions, e.g. from South Africa, and to avoid the dissemination of new and aggressive genotypes. Nevertheless, a link has been identified between the pathways of introduction and ultimate invasion success [66], since organismal traits that facilitate invasion by specific pathways may bestow success during the subsequent steps of introduction [67]. In addition, studies of introduction will help to pinpoint the centre of genetic diversity of the introduced species, and therefore significantly contribute to the search for effective biological control agents of invasive species. In this study, most of the Hap 4 (7 out of 8) that is most abundant in southern Australia are also found in southern Spain, where future biocontrol agents may be optimally sourced.

### Historical and recent genetic structure of *E*. *plantagineum* in the UK

Although *E*. *plantagineum* was reportedly cultivated as an ornamental plant in the UK since 1658 [23], it has displayed a very limited distribution in wild records from the 19^th^ century through to the present. The low level of genetic diversity observed in UK populations (Table 1) may be associated with small sample size, in association with limited distribution, but could also be indicative of the actual genetic structure of *E*. *plantagineum* in the UK. Genetically, historical UK herbarium samples collected since 1800 were very similar to current samples from naturalised populations in Cornwall. Only four haplotypes were observed among the historical *Echium* samples collected mainly from the southern UK. Of those, Hap 4 was observed at an 80% frequency, similar to that of this haplotype in the extant Cornish population (S2 Fig).

Taxonomists and botanists in the UK have long speculated whether *E*. *plantagineum* is native to the UK and Channel Islands. Results from this study show that genetic structure of *E*. *plantagineum* currently is very similar to that observed from historic samples collected since the mid 1800’s (pairwise *F*_*ST*_ = -0.049, *P* = 0.78), despite the fact that *E*. *plantagineum* has a minimal and restricted distribution in the UK. It is possible that Hap 4 was widely cultivated/naturalised as an ornamental species in the UK, followed by scattered introduction events of several additional haplotypes. What is clear from our results is that the UK is not a reservoir of genetic diversity for *E*. *plantagineum*. Low genetic diversity and absence of exclusive haplotypes do not support the presence of the species in the region over a long evolutionary time frame. In any case, it remains an open question as to whether some British populations originated from a relatively recent natural expansion from the mainland or are the result of human-mediated colonization. Additional research focused on the phylogeography and arrival of the species to the UK could properly address this question.

## Conclusion

Our findings demonstrate the important role of both UK and South African populations in shaping the genetic structure of the invasive weed species *E*. *plantagineum*, or Paterson’s curse, in Australia. Apart from proactive introduction as an ornamental plant in the 1840’s, accidental introduction associated with livestock movement and transport also appeared to play a critical role in the spread of this species across Australia. The high level of diversity detected in Australia is an indication that genetic bottleneck events (induced either by founder effects or selective sweeps) are unlikely to have affected the effective population size of the weed in Australia either during or after its introduction into the country. This suggests that environmental conditions in regions of establishment in Australia are broadly favourable to the sustained continuation of the species, creating the possibility of future spread and adaptation across Australia. Although our results evince multiple introduction pathways of the species to Australia, it would be still interesting to intensify the sampling both in the native and anciently introduced ranges across the Mediterranean basin and the Macaronesian archipelagos to unravel the distribution of haplotypes in the region and to assess the current occurrence of the six Australian/South African haplotypes undetected in our study in the Iberian native range. Our work offers a framework for further studies on the evolutionary processes involved in the invasiveness of the species, to address the question of whether pre-introduction adaptation/selection has occurred in populations from the UK or South Africa, which may have accelerated the invasion of Australia by *E*. *plantagineum*.

## Supporting information

S1 Table *Echium plantagineum* samples collected for DNA sequencing analysis, including year of collection, locality, GPS coordinates and GenBank accession number. An additional 129 samples from Australia (accession numbers KX012236-KX012622) sequenced in our previous study^23^ were also included in this study.
(DOCX)

S2 Table Primers used in this study. M13-vector sequences (underlined) were added to the 5’ end of forward and reverse primers to simplify the sequencing process.
(DOCX)

S3 Table *Echium plantagineum* haplotypes present in the native range (Iberian Peninsula), Australia, South Africa and the UK. *nh*: number of haplotypes found in each region. * indicates the presence of particular haplotype.
(DOCX)

S1 Fig Possible introduction pathway (arrows) of *E*. *plantagineum* to Australia implied from molecular markers in this study. This map is a derivative of “A large blank world map with oceans marked in blue.PNG” sourced from Wikimedia Commons, used under Public Domain license and modified using Adobe Illustrator CS5.
(TIF)

S2 Fig Abundance of different haplotypes in the UK inferred from historical samples (collected from 1845–1959) and the current samples. (TIF)

S1 Appendix Sequence alignment used in this study. (ZIP)
