# Supplementary material for: Genetic evidence for plural introduction pathways of the invasive weed Paterson’s curse (Echium plantagineum L.) to southern Australia
Source: PLoS One. 2019 Sep 19;14(9):e0222696. doi: 10.1371/journal.pone.0222696 (PMC6752891; doi:10.1371/journal.pone.0222696)
Supplement: S1 Table — An additional 129 samples from Australia (accession numbers KX012236-KX012622) sequenced in our previous study23 were also included in this study. (DOCX) [file pone.0222696.s001.docx]

S1 Table. *Echium plantagineum* samples collected for DNA sequencing analysis, including year of collection, locality, GPS coordinates and GenBank accession number. An additional 129 samples from Australia (accession numbers KX012236-KX012622) sequenced in our previous study^23^ were also included in this study.

| Sample ID | Year of collection | Country | Nearest locality | Latitude (S) | Longitude (E) | GenBank accession number | | |
| --- | --- | --- | --- | --- | --- | --- | --- | --- |
|  |  |  |  |  |  | *trnL* intron | *trnL*-*trnF* spacer | *trnH*-*psbA* spacer |
| ww19551 | 2013 | Australia | Wagga Wagga | 35.0581 | 147.352 | MG597841 | MG597967 | MG598181 |
| ww19624‡ | 1969 | Australia | Mallala | 34.45 | 138.48 | MG597842 | MG597968 | MG598182 |
| ww20612 | 2014 | Spain | Tarifa | -36.07 | -5.6925 | MG597843 | MG597969 | MG598183 |
| ww20614 | 2014 | Spain | Bolonia | -36.094 | -5.7795 | MG597844 | MG597970 | MG598184 |
| ww20616 | 2014 | Spain | Jerez | -36.639 | -6.084 | MG597845 | MG597971 | MG598185 |
| ww20617 | 2014 | Spain | Jerez | -36.639 | -6.084 | MG597846 | MG597972 | MG598186 |
| ww20618 | 2014 | Spain | Vigo | -42.167 | -8.6861 | MG597847 | MG597973 | MG598187 |
| ww20619 | 2014 | Spain | Vigo | -42.167 | -8.6861 | MG597848 | MG597974 | MG598188 |
| ww20624 | 2014 | Spain | Melide | -42.924 | -8.0673 | MG597849 | MG597975 | MG598189 |
| ww20626 | 2014 | Spain | Melide | -42.924 | -8.0673 | MG597850 | MG597976 | MG598190 |
| ww20627 | 2014 | Portugal | Areeiro | -40.188 | -8.4012 | MG597851 | MG597977 | MG598191 |
| ww20628 | 2015 | Portugal | Areeiro | -40.188 | -8.4012 | MG597852 | MG597978 | MG598192 |
| ww20632 | 2015 | Portugal | Pampilhosa do Botão | -40.313 | -8.4253 | MG597853 | MG597979 | MG598193 |
| ww20639 | 2015 | Portugal | Lousã | -40.022 | -8.221 | MG597854 | MG597980 | MG598194 |
| ww20640 | 2015 | Portugal | Lousã | -40.022 | -8.221 | MG597855 | MG597981 | MG598195 |
| ww20643 | 2015 | Portugal | Figueira da Foz | -40.16 | -8.676 | MG597856 | MG597982 | MG598196 |
| ww20647 | 2015 | Spain | Toledo | -39.86 | -4.03 | MG597857 | MG597983 | MG598197 |
| ww20648 | 2015 | Spain | Toledo | -39.86 | -4.03 | MG597858 | MG597984 | MG598198 |
| ww20660 | 2015 | Spain | El Casar | -40.7 | -3.43 | MG597859 | MG597985 | MG598199 |
| ww20661 | 2015 | Spain | El Casar | -40.7 | -3.43 | MG597860 | MG597986 | MG598200 |
| ww20662 | 2015 | Spain | Guadalix de la Sierra | -40.79 | -3.69 | MG597861 | MG597987 | MG598201 |
| ww20663 | 2015 | Spain | Guadalix de la Sierra | -40.79 | -3.69 | MG597862 | MG597988 | MG598202 |
| ww20666 | 2015 | Spain | Collado-Villalba | -40.63 | -4 | MG597863 | MG597989 | MG598203 |
| ww20668 | 2015 | Spain | Trescasas | -40.96 | -4.04 | MG597864 | MG597990 | MG598204 |
| ww20669 | 2015 | Spain | Trescasas | -40.96 | -4.04 | MG597865 | MG597991 | MG598205 |
| ww20672 | 2014 | Spain | Segovia | -40.95 | -4.11 | MG597866 | MG597992 | MG598206 |
| ww20673 | 2014 | Spain | Segovia | -40.95 | -4.11 | MG597867 | MG597993 | MG598207 |
| ww20674 | 2014 | Spain | San Juan de la Nava | -40.48 | -4.68 | MG597868 | MG597994 | MG598208 |
| ww20676 | 2014 | Spain | Fresnedilla | -40.23 | -4.62 | MG597869 | MG597995 | MG598209 |
| ww20677 | 2014 | Spain | Fresnedilla | -40.23 | -4.62 | MG597870 | MG597996 | MG598210 |
| ww20685 | 2014 | Portugal | Coimbra | -40.2 | -8.42 | MG597871 | MG597997 | MG598211 |
| ww20686 | 2014 | Portugal | Coimbra | -40.2 | -8.42 | MG597872 | MG597998 | MG598212 |
| ww20688 | 2014 | Portugal | Coimbra | -40.2 | -8.42 | MG597873 | MG597999 | MG598213 |
| ww21253* | 1886 | UK | Cornwall |  |  | - | - | - |
| ww21254* | 1957 | UK | Isles of Scilly |  |  | - | - | - |
| ww21255* | 1894 | UK | Cornwall |  |  | MG597874 | MG598000 | MG598214 |
| ww21256* | 1872 | UK | Cornwall |  |  | MG597875 | MG598001 | MG598215 |
| ww21257* | 1930 | UK | Cornwall |  |  | - | MG598002 | MG598216 |
| ww21258* | 1847 | UK | Isles of Wight |  |  | MG597876 | MG598003 | MG598217 |
| ww21259* | 1918 | UK | Hampshire |  |  | MG597877 | MG598004 | MG598218 |
| ww21260* | 1905 | UK | Isles of Jersey |  |  | MG597878 | MG598005 | MG598219 |
| ww21261* | 1867 | UK | Isles of Jersey |  |  | - | - | MG598220 |
| ww21262* | 1867 | UK | St Helens |  |  | - | - | - |
| ww21263* | 1926 | UK | Isles of Jersey |  |  | - | - | - |
| ww21264* | 1926 | UK | Isles of Jersey |  |  | MG597879 | MG598006 | MG598221 |
| ww21265* | 1923 | UK | Isles of Jersey |  |  | - | - | - |
| ww21266* | 1923 | UK | Isles of Jersey |  |  | - | MG598007 | - |
| ww21267* | 1938 | UK | Isles of Jersey |  |  | - | - | - |
| ww21268* | 1926 | UK | Hastings |  |  | - | - | - |
| ww21269* | 1886 | UK | Guernsey |  |  | MG597880 | MG598008 | MG598222 |
| ww21270* | 1959 | UK | Hampshire |  |  | MG597881 | MG598009 | MG598223 |
| ww21271* | 1953 | UK | Sussex |  |  | MG597882 | MG598010 | MG598224 |
| ww21272* | 1948 | UK | Surrey |  |  | - | - | - |
| ww21273* | 1972 | UK | Bedfordshire |  |  | - | - | - |
| ww21274* | 1961 | UK | Worcestershire |  |  | MG597883 | MG598011 | MG598225 |
| ww21275* | 1960 | UK | Worcestershire |  |  | - | - | - |
| ww21276* | 1910 | UK | Glamorganshire |  |  | - | - | - |
| ww21277* | 1960 | UK | Yorkshire |  |  | MG597884 | MG598012 | MG598226 |
| ww21278* | 1964 | UK | Yorkshire |  |  | MG597885 | MG598013 | MG598227 |
| ww21279* | 1964 | UK | Selkirkshire |  |  | MG597886 | MG598014 | MG598228 |
| ww21280* | 1963 | UK | Selkirkshire |  |  | - | - | - |
| ww21281* | 1967 | UK | Isle of Mull |  |  | - | - | - |
| ww21282* | 1884 | UK | Isles of Jersey |  |  | - | - | - |
| ww21283* | 1884 | UK | Isles of Jersey |  |  | - | - | - |
| ww21284* | 1889 | UK | Isles of Jersey |  |  | MG597887 | MG598015 | - |
| ww21285* | 1867 | UK | Isles of Jersey |  |  | MG597888 | MG598016 | MG598229 |
| ww21286* | 1845 | UK | Isles of Jersey |  |  | MG597889 | MG598017 | MG598230 |
| ww21287* | 1853 | UK | Isles of Jersey |  |  | - | - | - |
| ww21288* | 1858 | UK | Isles of Jersey |  |  | - | - | - |
| ww21289* | 1896 | UK | Isles of Jersey |  |  | - | - | - |
| ww21290* | 1938 | UK | Isles of Jersey |  |  | - | - | - |
| ww21291* | 1930 | UK | Isles of Jersey |  |  | MG597890 | MG598018 | - |
| ww21292* | 1930 | UK | Isles of Jersey |  |  | - | - | - |
| ww21336 | 2015 | Spain | El Burgo de Ardales | -36.84 | -4.8894 | MG597891 | MG598019 | MG598231 |
| ww21337 | 2015 | Spain | El Burgo de Ardales | -36.84 | -4.8894 | MG597892 | MG598020 | MG598232 |
| ww21338 | 2015 | Spain | El Burgo de Ardales | -36.84 | -4.8894 | MG597893 | MG598021 | MG598233 |
| ww21339 | 2015 | Spain | Villaluenga del Rosario | -36.695 | -5.3841 | MG597894 | MG598022 | MG598234 |
| ww21340 | 2015 | Spain | Villaluenga del Rosario | -36.695 | -5.3841 | MG597895 | MG598023 | MG598235 |
| ww21831 | 2015 | Spain | Villaluenga del Rosario | -36.695 | -5.3841 | MG597896 | MG598024 | MG598236 |
| ww21832 | 2015 | Spain | Villaluenga del Rosario | -36.695 | -5.3841 | MG597897 | MG598025 | MG598237 |
| ww21833 | 2015 | Spain | Villaluenga del Rosario | -36.695 | -5.3841 | MG597898 | MG598026 | MG598238 |
| ww21835 | 2015 | Spain | Cadiz | -36.535 | -6.297 | MG597899 | MG598027 | MG598239 |
| ww21836 | 2015 | Spain | Cadiz | -36.535 | -6.297 | MG597900 | MG598028 | MG598240 |
| ww21837 | 2015 | Spain | Cadiz | -36.535 | -6.297 | MG597901 | MG598029 | MG598241 |
| ww21838 | 2015 | Spain | Cadiz | -36.535 | -6.297 | MG597902 | MG598030 | MG598242 |
| ww21841 | 2015 | South Africa | Cape Town | 34.1343 | 18.9012 | MG597903 | MG598031 | MG598243 |
| ww21842 | 2015 | South Africa | Cape Town | 34.1343 | 18.9012 | MG597904 | MG598032 | MG598244 |
| ww21843 | 2015 | South Africa | Cape Town | 34.1343 | 18.9012 | MG597905 | MG598033 | MG598245 |
| ww21844 | 2015 | South Africa | Cape Town | 34.1343 | 18.9012 | MG597906 | MG598034 | MG598246 |
| ww21845 | 2015 | South Africa | Cape Town | 34.1343 | 18.9012 | MG597907 | MG598035 | MG598247 |
| ww21846 | 2015 | South Africa | Cape Town | 34.1453 | 18.8547 | MG597908 | MG598036 | MG598248 |
| ww21847 | 2015 | South Africa | Cape Town | 34.1453 | 18.8547 | MG597909 | MG598037 | MG598249 |
| ww21848 | 2015 | South Africa | Cape Town | 34.1453 | 18.8547 | MG597910 | MG598038 | MG598250 |
| ww21849 | 2015 | South Africa | Cape Town | 34.1453 | 18.8547 | MG597911 | MG598039 | MG598251 |
| ww21850 | 2015 | South Africa | Cape Town | 34.1453 | 18.8547 | MG597912 | MG598040 | MG598252 |
| ww21851 | 2015 | South Africa | Cape Town | 34.0728 | 18.8182 | MG597913 | MG598041 | MG598253 |
| ww21852 | 2015 | South Africa | Cape Town | 34.0728 | 18.8182 | MG597914 | MG598042 | MG598254 |
| ww21853 | 2015 | South Africa | Cape Town | 34.0728 | 18.8182 | MG597915 | MG598043 | MG598255 |
| ww21854 | 2015 | South Africa | Cape Town | 34.0728 | 18.8182 | MG597916 | MG598044 | MG598256 |
| ww21855 | 2015 | South Africa | Cape Town | 34.0728 | 18.8182 | MG597917 | MG598045 | MG598257 |
| ww21856 | 2015 | South Africa | Cape Town | 34.0377 | 18.7257 | MG597918 | MG598046 | MG598258 |
| ww21857 | 2015 | South Africa | Cape Town | 34.0377 | 18.7257 | MG597919 | MG598047 | MG598259 |
| ww21858 | 2015 | South Africa | Cape Town | 34.0377 | 18.7257 | MG597920 | MG598048 | MG598260 |
| ww21859 | 2015 | South Africa | Cape Town | 34.0377 | 18.7257 | MG597921 | MG598049 | MG598261 |
| ww21860 | 2015 | South Africa | Cape Town | 34.0377 | 18.7257 | MG597922 | MG598050 | MG598262 |
| ww21861 | 2015 | South Africa | Stellenbosch | 33.9903 | 18.8051 | MG597923 | MG598051 | MG598263 |
| ww21862 | 2015 | South Africa | Stellenbosch | 33.9903 | 18.8051 | MG597924 | MG598052 | MG598264 |
| ww21863 | 2015 | South Africa | Stellenbosch | 33.9903 | 18.8051 | MG597925 | MG598053 | MG598265 |
| ww21864 | 2015 | South Africa | Stellenbosch | 33.9903 | 18.8051 | MG597926 | MG598054 | MG598266 |
| ww21865 | 2015 | South Africa | Stellenbosch | 33.9903 | 18.8051 | MG597927 | MG598055 | MG598267 |
| ww21866 | 2015 | South Africa | Stellenbosch | 33.9568 | 18.8593 | MG597928 | MG598056 | MG598268 |
| ww21867 | 2015 | South Africa | Stellenbosch | 33.9568 | 18.8593 | MG597929 | MG598057 | MG598269 |
| ww21868 | 2015 | South Africa | Stellenbosch | 33.9568 | 18.8593 | MG597930 | MG598058 | MG598270 |
| ww21869 | 2015 | South Africa | Stellenbosch | 33.9568 | 18.8593 | MG597931 | MG598059 | MG598271 |
| ww21870 | 2015 | South Africa | Stellenbosch | 33.9568 | 18.8593 | MG597932 | MG598060 | MG598272 |
| ww21871 | 2015 | South Africa | Caledon | 34.2294 | 19.5247 | MG597933 | MG598061 | MG598273 |
| ww21872 | 2015 | South Africa | Caledon | 34.2294 | 19.5247 | MG597934 | MG598062 | MG598274 |
| ww21873 | 2015 | South Africa | Caledon | 34.2294 | 19.5247 | MG597935 | MG598063 | MG598275 |
| ww21874 | 2015 | South Africa | Caledon | 34.2294 | 19.5247 | MG597936 | MG598064 | MG598276 |
| ww21875 | 2015 | South Africa | Caledon | 34.2294 | 19.5247 | MG597937 | MG598065 | MG598277 |
| ww21876 | 2015 | South Africa | Cape Town | 33.7765 | 18.7409 | MG597938 | MG598066 | MG598278 |
| ww21877 | 2015 | South Africa | Cape Town | 33.7765 | 18.7409 | MG597939 | MG598067 | MG598279 |
| ww21878 | 2015 | South Africa | Cape Town | 33.7765 | 18.7409 | MG597940 | MG598068 | MG598280 |
| ww21879 | 2015 | South Africa | Cape Town | 33.7765 | 18.7409 | MG597941 | MG598069 | MG598281 |
| ww21880 | 2015 | South Africa | Cape Town | 33.7765 | 18.7409 | MG597942 | MG598070 | MG598282 |
| ww21881 | 2015 | South Africa | Cape Town | 33.957 | 18.4819 | MG597943 | MG598071 | MG598283 |
| ww21882 | 2015 | South Africa | Cape Town | 33.957 | 18.4819 | MG597944 | MG598072 | MG598284 |
| ww21883 | 2015 | South Africa | Cape Town | 33.957 | 18.4819 | MG597945 | MG598073 | MG598285 |
| ww21884 | 2015 | South Africa | Cape Town | 33.957 | 18.4819 | MG597946 | MG598074 | MG598286 |
| ww21885 | 2015 | South Africa | Cape Town | 33.957 | 18.4819 | MG597947 | MG598075 | MG598287 |
| ww21886 | 2015 | South Africa | George | 33.9585 | 22.3823 | MG597948 | MG598076 | MG598288 |
| ww21887 | 2015 | South Africa | George | 33.9585 | 22.3823 | MG597949 | MG598077 | MG598289 |
| ww21888 | 2015 | South Africa | George | 33.9585 | 22.3823 | MG597950 | MG598078 | MG598290 |
| ww21889 | 2015 | South Africa | George | 33.9585 | 22.3823 | MG597951 | MG598079 | MG598291 |
| ww21890 | 2015 | South Africa | George | 33.9585 | 22.3823 | MG597952 | MG598080 | MG598292 |
| ww21891 | 2016 | UK | Kelynack | -50.11 | -5.6944 | MG597953 | MG598081 | MG598293 |
| ww21892 | 2016 | UK | Kelynack | -50.11 | -5.6944 | MG597954 | MG598082 | MG598294 |
| ww21893 | 2016 | UK | Kelynack | -50.11 | -5.6947 | MG597955 | MG598083 | MG598295 |
| ww21894 | 2016 | UK | Kelynack | -50.11 | -5.6947 | MG597956 | MG598084 | MG598296 |
| ww21895 | 2016 | UK | Kelynack | -50.11 | -5.6961 | MG597957 | MG598085 | MG598297 |
| ww21896 | 2016 | UK | Kelynack | -50.11 | -5.6961 | MG597958 | MG598086 | MG598298 |
| ww21897 | 2016 | UK | Kelynack | -50.109 | -5.6953 | MG597959 | MG598087 | MG598299 |
| ww21898 | 2016 | UK | Kelynack | -50.109 | -5.6953 | MG597960 | MG598088 | MG598300 |
| ww21899 | 2016 | UK | Kelynack | -50.108 | -5.6951 | MG597961 | MG598089 | MG598301 |
| ww21900 | 2016 | UK | Kelynack | -50.108 | -5.6951 | MG597962 | MG598090 | MG598302 |
| ww21901 | 2016 | Portugal |  |  |  | MG597963 | MG598091 | MG598303 |
| ww21902 | 2016 | Spain |  |  |  | MG597964 | MG598092 | MG598304 |
| ww21903 | 2016 | Spain |  |  |  | MG597965 | MG598093 | MG598305 |
| ww21904 | 2016 | Spain |  |  |  | MG597966 | MG598094 | MG598306 |

- indicates PCR or sequencing not successful, ‡ indicates samples donated by Australian National Herbarium, * indicates samples donated by British Natural History Museum
