# Supplementary material for: Genetic evidence for plural introduction pathways of the invasive weed Paterson’s curse (Echium plantagineum L.) to southern Australia
Source: PLoS One. 2019 Sep 19;14(9):e0222696. doi: 10.1371/journal.pone.0222696 (PMC6752891; doi:10.1371/journal.pone.0222696)
Supplement: S2 Table — M13-vector sequences (underlined) were added to the 5’ end of forward and reverse primers to simplify the sequencing process. (DOCX) [file pone.0222696.s002.docx]

S2 Table. Primers used in this study. M13-vector sequences (underlined) were added to the 5’ end of forward and reverse primers to simplify the sequencing process.

| Gene | Primer Name | Sequence (5'-3') | T_A_ (ºC) | References* |
| --- | --- | --- | --- | --- |
| *trnH*-*psbA* | trnH | GTAAAACGACGGCCAGTACTGCCTTGATCCACTTGGC | 62 | ^38^ |
|  | psbA | CAGGAAACAGCTATGACCGAAGCTCCATCTACAAATGG |  | ^38^ |
| *trnL* intron | ucp_c | GTAAAACGACGGCCAGTCGAAATCGGTAGACGCTACG | 58 | ^37^ |
|  | ucp_d | CAGGAAACAGCTATGACGGGGATAGAGGGACTTGAAC |  | ^37^ |
| *trnL*-*trnF* spacer | ucp_e | GTAAAACGACGGCCAGTGGTTCAAGTCCCTCTATCCC | 64 | ^37^ |
|  | ucp_f | CAGGAAACAGCTATGACATTTGAACTGGTGACACGAG |  | ^37^ |

*Please refer to the main text for the references.
