# Supplementary material for: Genetic evidence for plural introduction pathways of the invasive weed Paterson’s curse (Echium plantagineum L.) to southern Australia
Source: PLoS One. 2019 Sep 19;14(9):e0222696. doi: 10.1371/journal.pone.0222696 (PMC6752891; doi:10.1371/journal.pone.0222696)
Supplement: S3 Table — nh: number of haplotypes found in each region. * indicates the presence of particular haplotype. (DOCX) [file pone.0222696.s003.docx]

S3 Table. *Echium plantagineum* haplotypes present in the native range (Iberian Peninsula), Australia, South Africa and the UK. *nh*: number of haplotypes found in each region. * indicates the presence of particular haplotype.

| Region | *nh* | H1 | H2 | H3 | H4 | H5 | H6 | H7 | H8 | H9 | H10 | H11 | H12 | H13 | H14 |
| --- | --- | --- | --- | --- | --- | --- | --- | --- | --- | --- | --- | --- | --- | --- | --- |
| Iberian Peninsula | 8 |  | * | * | * |  | * | * | * |  |  | * |  | * |  |
| Australia | 12 | * | * | * | * | * | * | * | * | * | * | * | * |  |  |
| South Africa | 12 | * | * | * | * | * | * | * | * | * |  | * | * |  | * |
| UK | 4 |  | * |  | * | * | * |  |  |  |  |  |  |  |  |
