# Supplementary figures and images for: Genetic evidence for plural introduction pathways of the invasive weed Paterson’s curse (Echium plantagineum L.) to southern Australia
Source: PLoS One. 2019 Sep 19;14(9):e0222696. doi: 10.1371/journal.pone.0222696 (PMC6752891; doi:10.1371/journal.pone.0222696)

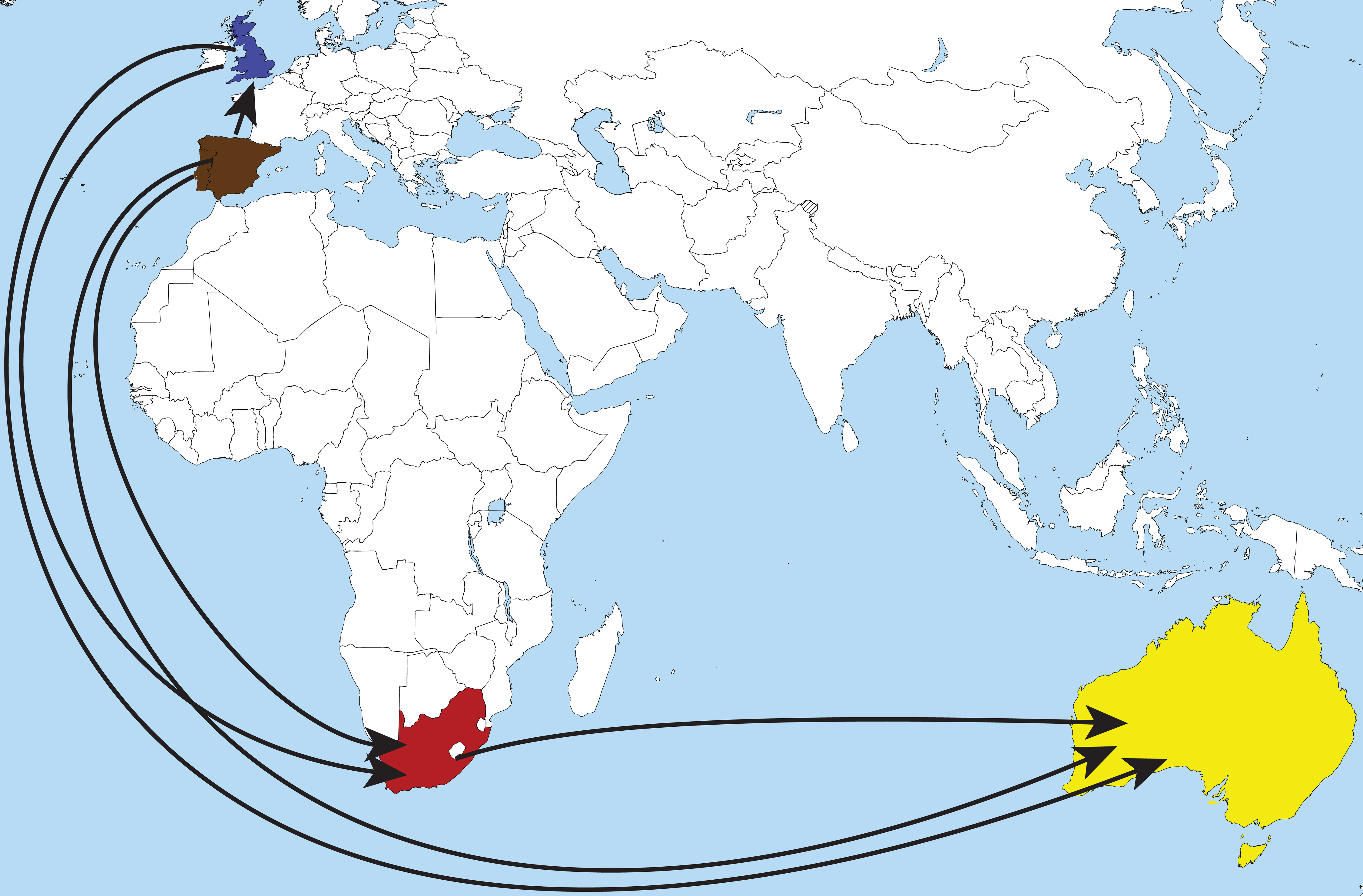

Supplement: S1 Fig — This map is a derivative of “A large blank world map with oceans marked in blue.PNG” sourced from Wikimedia Commons, used under Public Domain license and modified using Adobe Illustrator CS5. (TIF) [file pone.0222696.s004.tif]

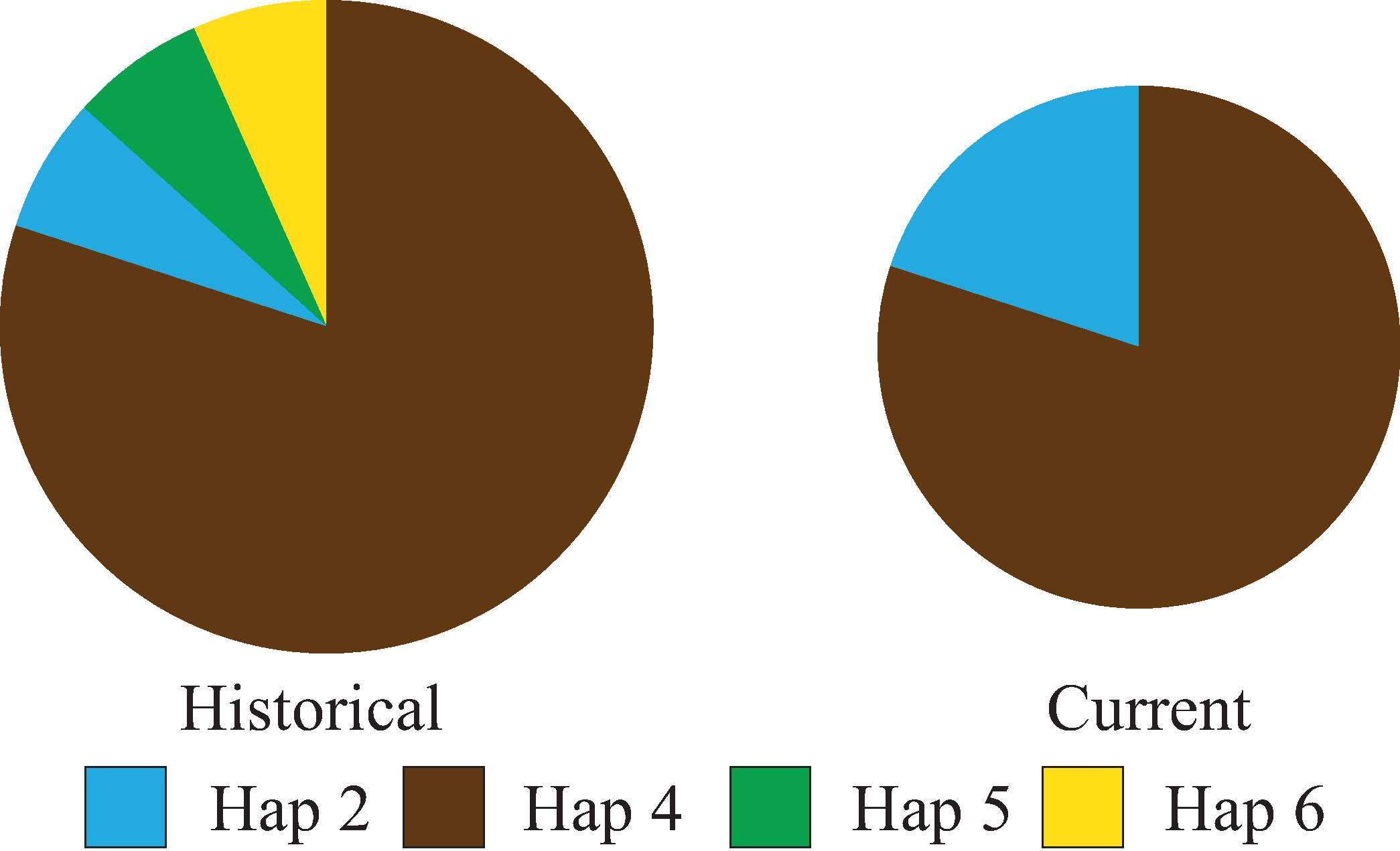

Supplement: S2 Fig — (TIF) [file pone.0222696.s005.tif]
